# Elicitation: A Tool for Enriching the Bioactive Composition of Foods

**DOI:** 10.3390/molecules190913541

**Published:** 2014-09-01

**Authors:** Nieves Baenas, Cristina García-Viguera, Diego A. Moreno

**Affiliations:** Phytochemistry Laboratory, Department of Food Science and Technology, CEBAS-CSIC, Campus de Espinardo, Edificio 25, 30100 Murcia, Spain; E-Mails: nbaenas@cebas.csic.es (N.B.); cgviguera@cebas.csic.es (C.G.-V.)

**Keywords:** elicitor, phytochemicals, health, phenolics, glucosinolates, activity

## Abstract

Elicitation is a good strategy to induce physiological changes and stimulate defense or stress-induced responses in plants. The elicitor treatments trigger the synthesis of phytochemical compounds in fruits, vegetables and herbs. These metabolites have been widely investigated as bioactive compounds responsible of plant cell adaptation to the environment, specific organoleptic properties of foods, and protective effects in human cells against oxidative processes in the development of neurodegenerative and cardiovascular diseases and certain types of cancer. Biotic (biological origin), abiotic (chemical or physical origin) elicitors and phytohormones have been applied alone or in combinations, in hydroponic solutions or sprays, and in different selected time points of the plant growth or during post-harvest. Understanding how plant tissues and their specific secondary metabolic pathways respond to specific treatments with elicitors would be the basis for designing protocols to enhance the production of secondary metabolites, in order to produce quality and healthy fresh foods.

## 1. Introduction: Secondary Metabolites in Plants, Foods and Human Health

Plant-based nutrients and phytochemicals present in vegetable foods include proteins, lipids, carbohydrates, vitamins, minerals, and bioactive compounds, including phenolic compounds and glucosinolates, that confer additional advantages to plant cell adaptation capacity to the surrounding environment, and act as precursors of molecules involved in the plant defense systems such as antibiotics, antifungals, and antivirals. Therefore, secondary metabolites are able to protect plants from pathogens (phytoalexins) [1] and insects [2], as well as constituting important UV-radiation absorbing compounds, thus preventing serious leaf damage [3]. The content of secondary metabolites in vegetables also confers a relevant role as health-promoting compounds and therefore contributes to their economic importance of foods [4]. Phenolic compounds contribute significantly to imparting specific flavours and colours to various plants widely utilized in foods and beverages. Examples includes capsaicin, responsible for the pungent properties of the red peppers, alkylphenols, responsible for the characteristic taste and odour of clove oil, tannins, which add a distinct bitterness or astringency to the taste of certain foods, and the anthocyanin pigments, such as the pelargonidins, the cyanidins and the delphinidins (responsible for red, blue and purple colours) [5]. The glucosinolates, characteristic of cruciferous foods, also add bitter taste (progoitrin) and aroma intensity (total glucosinolates) to vegetables [6].

The relevance of phenolic compounds [7] and glucosinolates [8] for human consumption has been associated with a protective effect against oxidative processes in relation to cardiovascular and central nervous system health, and neurodegenerative diseases, and with a reduced risk for cancers of the gastrointestinal tract, lung, colon, bladder, pancreas, skin, breast and prostate [9]. Optimizing the composition of fruits and vegetables would be a very cost-effective method for improving nutrition and disease prevention, since diet-induced health improvements would not represent any added costs for the health sector, even more it might help to reduce these costs [10,11,12].

The phytochemical composition of plants foods vary according to genetics (family, species, cultivar, *etc.*), physiological (organ, maturity and age) and agronomical factors (photoperiod, saline stress or fertilization) [13,14,15,16,17,18,19]. These factors are grouped as biotic (genetics, physiological determinants, pests and diseases) and abiotic (environment and agronomical conditions) and can be used to enhance valuable metabolites in foods and ingredients, in a year-round production [16,17,20]. Specific treatments, including precursor feeding and elicitor application can be used to increase metabolite production in the plant and to enhance its qualitative value for fresh produce, enriched food, or as a raw ingredient for feed/food and pharmaceutical products [21,22].

## 2. Elicitors

### 2.1. Concept and Classification

Elicitors are substances which induce physiological changes in the plant. Plants respond to these stressors by activating an array of mechanisms, similar to the defense responses to pathogen infections or environmental stimuli, affecting the plant metabolism and enhancing the synthesis of phytochemicals. The first biotic elicitors were described in the early 1970s [23]. Since then, numerous publications have accumulated evidence for pathogen-derived compounds that induce defense responses in intact plants [24,25] or plant cell cultures [22,26]. The use of elicitors as a tool to enhance the phytochemical content in plants, applied alone or in combinations at selected time points of the vegetable growth, should not be confused with those administered during the plant production cycle or pre-harvest, such as conventional fertilization.

Elicitors could be classified as biotic and abiotic compounds, also plant hormones (salicylic acid (SA), jasmonates, *etc.*) may be considered as elicitors (Table 1) [27,28].

**Table 1:** Elicitor classification based on their origin.

**Biotic Elicitors**	
Lipopolysaccharides [27]	
Polysaccharides: Pectin and cellulose (cell walls) [28]; chitosan [21,28], chitin and glucans (microorganisms) [28], alginate, arabic gum [29], guar gum, LBG [27], yeast extract [27].	
Oligosaccharides: Galacturonides, guluronate, mannan, mannuronate [27,30].	
Proteins: Cellulase [31], cryptogein [32], glycoproteins [27], oligandrin [27], pectolyase, fish protein hydrolysates [33], lactoferrin [33].	
Complex composition: Fungal spores, mycelia cell wall, microbial cell wall [27].	
Pathogen toxin: Coronatine [34].	
Oregano extract [33].	
**Abiotic Elicitors**	
Chemical	Physical [35]
Acetic acid [21]	Altered gas composition
Benzothiadiazole [36]	Chilling
Silicon [36]	CO_2_
Bioregulator prohexadione	Drought
Ethanol [37]	Extreme temperature shock
Ethene [37]	High pressure
Inorganic salts: mercuric chloride (HgCl2), copper sulfate (CuSO4), calcium chloride (CaCl2), and vanadyl sulfate (VSO4) [28]	High or low osmolarity UV irradiation Saline stress
Metal ions: Co^2+^, Fe^2+^, Al^3+^, Ag^2+^, Ag^+^, Mn^2+^, Zn^2+^, Cu^2+^, Pb^2+^ and Cd^2+^ [28,38]	Wounding Ozone
**Plant Hormones**	
Jasmonic acid, methyl jasmonate [39], methyl salicylate, salicylic acid, ethylene [21,40], cytokinin, gibberellin GA_3_ [37].	

Biotic elicitors (chitosan, alginate, cellulose, *etc.*) have biological origin, often originated as a result of fungi, bacteria, virus or herbivore infections (exogenous elicitors), and in some cases are released from the attacked plant by the action of enzymes of the pathogen (endogenous elicitors) [27]. Often complex biological preparations have been used as elicitors, where the molecular structure of the active ingredients is unknown. Examples of such elicitors are yeast extract and microbial cell-wall preparations [27]. Yeast extract contains several components that can elicit plant defense responses, including chitin, N-acetylglucosamine oligomers, β-glucan, glycopeptides and ergosterol.

SA and jasmonates (jasmonic acid (JA), methyl jasmonate (MeJA)) are widely known to elicit a wide range of compounds by inducing the expression of plant genes for various biosynthetic pathways, and are also defined as “hormones” because they induce cellular responses at low concentrations distant from their site of synthesis, and can be applied to plants in a variety of ways. For instance, MeJA may be applied to plants as a gas in an enclosed environment, on a liquid form to a hydroponic solution, or by jasmonate sprays [39]. The treatment of young red and black raspberry fruits with 0.01 mM or 0.1 mM MeJA increased their anthocyanins and phenolic compounds [41]. Analogs of MeJA or JA have physiological activity. For instance, N-propyl dihydrojasmonate (PDJ) increased the abscisic acid (ABA) and anthocyanin content of apples [42]. Abiotic elicitors are produced by factors responsible for environmental stress. These factors can be of chemical (inorganic salts, metal ions and others which disturb the membrane integrity) [28] and physical origin (UV irradiation, wounding, saline stress, ozone *etc.*) [35] (Table 1). For instance, exposure of alfalfa, broccoli and radish 3-old-day sprouts to high light intensity (700 µmol·m^−2^ s^−1^ for 1 day) or chilling (4 °C and and 120 µmol·m^−2^ s^−1^ for 1 day) resulted in higher total phenolic content and antioxidant capacity compared with controls, by 20% in alfalfa and 40% in broccoli, and showed a 25% increase of phenolic content and 40% of higher antioxidant capacity in radish [43].

Apart from the classification of elicitors according to their nature, they can also be classified upon their interaction with the host plant, as “general elicitors”, such as carbohydrates, cell wall proteins, oligosaccharides *etc.*, which induce non-specific mechanisms for the induction of defense response in different plant cultures, and “specific elicitors” from fungal, bacterial, viral or plant origin, which affect only a specific host cultivar since the presence of its corresponding resistance gene in the host plant is directly associated with the resistance against a specific gene pathogen [4,44].

### 2.2. Mode of Action of Elicitors

**Figure 1:**
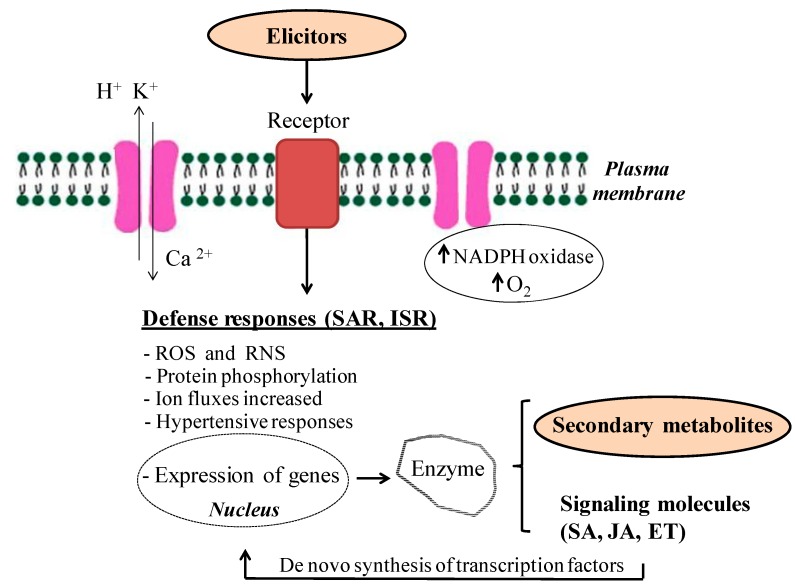
General mechanism after elicitor perception. Abbreviations: SAR (systemic adquired response), ISR (induced systemic resistance), ROS (Reactive oxygen species), RNS (reactive nitrogen species), NADPH (nicotinamide adenine dinucleotide phosphate), SA (salicylic acid), JA (jasmonic acid), ET (ethylene) [48,49,50].

In plant defense systems each cell has acquired the capability to respond to pathogens and environmental stresses and to build up a defense response. Plant response is determined by several factors, mainly depending on their genetic characteristics and physiological state. In the majority of cases, plant resistance to diseases is known to be genetically controlled by plant resistance (R) genes and pathogen avirulent avirulence (Avr) genes (gene-for-gene interaction concept) [45]. However, triggering resistance is not always due to specific Avr products which activate defense responses in cultivars possessing the matching R genes but, instead, proceeds from the action of general elicitors, able to activate defenses in different cultivars of one or many species [45]. First step in the response of plant against elicitors is the stimulus perception by receptors localized in plasma membranes of the plant cell (Figure 1), like protein kinases, which represent one of the most important in pathogen perception for a number of fungal elicitors [46], or could be localized within the cell to initiate signaling processes that activate plant defenses, as for certain bacterial elicitors, which initiate signaling processes that activate plant defenses [47].

The elicitor signal transduction is an important subject of investigation. In this sense, several authors have described that plants respond to elicitors by activating an array of defense mechanisms on the surface of the plasma membrane (Figure 1), including induction of pathogenesis-related proteins and enzymes of oxidative stress protection, hypertensive responses, characterized by rapid cell death in the immediate vicinity of the point of exposure to the pathogen [45], the production of reactive oxygen species (ROS) and reactive nitrogen species (RNS), the activation of defense-related genes, changes in the potential of plasma membrane cell and enhanced ion fluxes (Cl^−^ and K^+^ efflux and Ca^2+^ influx), rapid changes in protein phosphorylation, lipid oxidation, and structural defensive barriers, such as reinforcement and lignification deposition inn cell wall, *etc.* and the activation and the *de novo* biosynthesis of transcription factors, which directly regulate the expression of genes involved in secondary metabolites production [48,49,50] (Figure 1).

### 2.3. Preharvest Elicitation: Priming Seeds and Edible Plants

Preharvest elicitation could be done as seed priming [33,51], soaking seeds in a water solution with the elicitor, or after seedling, applying exogenous spraying treatment over the leaves [52] or in a hydroponic system [53].

**Figure 2:**
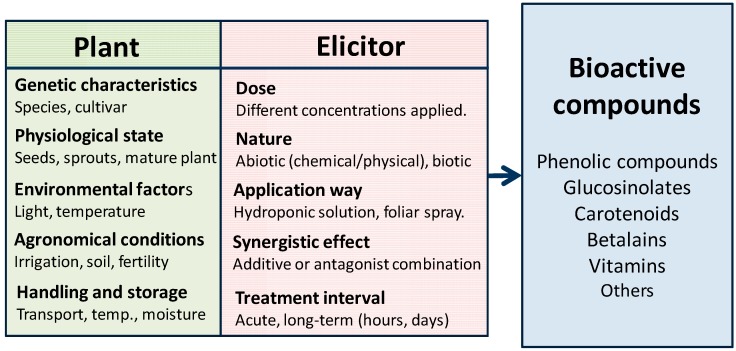
Factors influencing bioactive compounds in plant response.

Elicitor nature, doses and time of treatment strongly affects the intensity of the plant response (Figure 2). Elicitors can stimulate different classes of secondary metabolites and affect in a different way the concentration of these compounds, being more dependent on plant genetics (species and cultivars) than on the elicitor nature.

A MeJA elicitation, applied daily by exogenous spraying at 10 µM, resulted in a 31%, 23% and 22% increase of total flavonoid, phenolic and glucosinolates concentration, respectively, in 7 day old broccoli sprouts [25]. Also a MeJA sprayed treatment (10 mM) at the beginning of veraison in grape (*Vitis vinifera*) increased anthocyanin and flavonols content up to 81% and 131%, respectively [54].

Concentration of elicitor and interval between treatment and harvest induce different responses characteristic of plant species, making necessary to find the adequate effective dose and time empirically [4]. Radish sprouts (*Raphanus sativus* L.) treated with 100 mM of NaCl increased total glucosinolates in 5- and 7-day-old sprouts, by 50% and 127%, respectively, and the phenolic contents in 3- and 5-day-old sprouts, by 20% and 40%, respectively, while with a low and moderate level of salt stress (10–50 mM of NaCl) reduced these contents [55]. Bodnaryk showed that JA and MeJA were equally effective at high doses (>5 nmol seedling^−1^) in increasing the concentration of 3-indolylmethyl glucosinolates (3-IMG), maybe because of the saturated effect of jasmonates, but at lower doses, JA was more potent than MeJA [56]. The dose needed to cause a doubling of the concentration of 3-IMG in the cotyledons of 7-day-old *B. napus* sprouts, in 24 hs, was 8.2 pmol for JA and 41 pmol for MeJA. The sulphur effect, as elicitor, in broccoli sprouts was dependent on the dosage (K_2_SO_4_ at 15, 30, and 60 mg/L) and augmented the total glucosinolates in sprouts by 14%, 18%, and 23%, respectively, 12 days after sowing [57].

Physiological conditions also play an important role in the elicitation techniques, which achieving better results during the exponential phase of growth of the plant, when the concentration of bioactive compounds is higher [58], and in the presence of growth regulators [59].

Different studies have reported an additive or synergistic response after combination of elicitor treatments, different signal transduction pathways appear to exist in response to environmental stresses and elicitors and these pathways could antagonize or harmonize with each other, leading to negative or additive interactions, respectively [58,60,61].

### 2.4. Postharvest Elicitors Applications

Specific elicitor treatments has been used in postharvest practices to enhance the phytochemical content and quality composition in many fruits and vegetables, such as the application of low or high temperature treatments [62], ultraviolet (UV) [63,64] or gas combinations before commercialization [40]. In this context, it has to be mentioned that red orange fruits (*Citrus sinensis*) accumulated anthocyanins (8-fold compared to control) in their juice vesicles during cold storage at 4 °C for a period of 75 days [62]. An accumulation of phenolic compounds was also found in apple (*Malus domestica*) during cold storage which was coupled with increasing the phenylalanine ammonialyse (PAL) activity, a key enzyme in the phenylpropanoid pathway [65]. A combination of visible light and UV-B irradiation (380 nm) applied 12 h per day during a period of 10 days, increased the total phenolic compounds (127% compared to irradiation of visible light alone) in apple peel. It was assumed that UV-stress also mediated the increase of PAL activity [64]. Ultraviolet irradiation can lead to grapes with enhanced antioxidant properties, within normal conditions of market commercialization [63].

On the other hand, phytohormones applied to tissues will increase phenolic concentration. For instance, ethylene applied to butter leaf lettuce at 10 µL·L^−1^ in a flow of humid air for 3 days at 5 °C, induced synthesis of phenolic compounds by 38%, even though wounding increased by 87% these compounds [40]. Furthermore, the authors observed that temperature also affected the concentration of phenolics, at 10 °C ethylene and wounding induced increases of 174% and 155%, respectively. The exogenous application of the phytohormone MeJA (170 µL spontaneously vaporized at 25 °C) over strawberry fruits during 7 days, induced an increase of 35%, 52% and 187%, on phenolic content, antioxidant capacity, and anthocyanins, respectively [66]. A longer storage, after 11 days, resulted in a considerable decline of total phenolic content and antioxidant capacity, detrimental of fruit quality. On the other hand, through elicitor practices also the quality of food products could be enhance, such as the improvement of the volatile profile, flavor and taste of wine after a chitosan treatment or the increase of phenolic compounds of peppermint resulting infusions after SA foliar application in the plant [67,68]. Understanding the interactions among the stressor applied and the tissue response will help to optimize the right application.

Alternatively to a hierarchical response, additive or synergistic responses can be used to selectively target the increase of bioactive compounds [21,69]. Synergistic effects have also been found for postharvest elicitors, in sorghum seedlings exposed to low moderate temperatures during 24 h before a red light irradiation by fluorescent tubes (661 nm), resulting the optimum temperature at 20 °C for enhancement of red light induced anthocyanin synthesis (185%) compared that for seedlings growth at 24 °C [70]. The use of wounding (3 mm thick disks sliced) in combination with ethylene (1000 ppm) and MeJA (250 ppm) in purple carrot (*Daucus carota* L.) increased the total phenolic content by about 176% and 210%, respectively, compared to the separate treatments [71].

## 3. Elicitation Effects on Primary Metabolism

Plant primary metabolism includes physical and chemical processes that fulfill the essential functions for the maintenance of plant life: survival, growth and reproduction. Photosynthesis, respiration, nutrient uptake, transport and partitioning, protein synthesis, tissue differentiation, biosynthesis of carbohydrates, lipids and the proteins involved in these processes or in structural parts are all chemical processes belonging to the primary metabolism. Biotic and abiotic stresses (variation in agronomical conditions, such as plant organ, plant competition, fertilization, pH, season, climate, water availability, light, and CO_2_ [9]) are expressed in plants by a series of morphological, physiological, biochemical and molecular changes that adversely affect plant growth and productivity [72].

Gómez *et al.*, studied MeJA spray application (0.5 mM) to the foliage of tomato plants for 4 h. There was a significant decrease in the fixation of CO_2_ (20%) and an increase in the export of newly acquired carbon and nitrogen (1-fold) out of MeJA-treated leaves [73]. These results showed a change in the allocation of resources after MeJA application, this may reduce the chance of resources being lost to herbivores and act as a buffer to biotic stress by increasing the potential for plant regrowth and survival after the attack.

The effects on the germination of alfalfa and broccoli seeds stimulated by dry smoke (by the complete combustion of *Artemisia vulgaris*) during 30 and 45 min, respectively, and aspirin solution (0.145 g/100 mL in pure water) during 10 and 30 min, respectively, showed higher growth ratio than control group (>112%) [74].

A treatment of chitosan (28 kDa), a deacetylated derivative of chitin, at 0.5% dissolved in 0.5% lactic acid, increased the total weight (12.9%), germination rate (16%) and total isoflavone content (11.8%) of sunflower sprouts [51], while a treatment in soybean sprouts with 0.05% chitosan (493 kDa) in 0.05% acetic acid solution increased the total weight (26%) and vitamin C content (14%) compared with that of the control [51,75].

Baenas *et al.*, showed an increase in biomass weight of 5 different *Brassicaceae* sprouts after 5-days spray elicitation with sucrose (146 mM), as a supply of carbon source for cell growth, and DL-methionine (5 mM), enhancing the overexpression of some genes [52].

## 4. Elicitors Affecting the Content of Bioactive Compounds

The most actively pursued strategies to increase the production of target natural products in plants, are the applications of chemical elicitors and the study of the signal transduction pathways and transcription factors required for the expression of genes, involved in the biosynthesis of specific bioactive phytochemicals [50].

Much effort has been put into cloning biosynthetic genes, identifying transcription factors, revealing the signal transduction steps underlying elicitor activation of plant secondary metabolism and also into the manipulation of regulatory and biosynthetic genes, to engineer plant cells and enhance the production of target secondary metabolites [76]. It is expected that a better understanding of the signal transduction pathways, linking plant cell stimulation and biosynthesis of natural compounds may help to develop new strategies to alter the production of target compounds, by either activation or suppression of certain metabolic pathways [48]. As a consequence, in plant tissues is observed the production of antioxidant molecules, compounds of technological interest in healthy foods [48]. Hao *et al.*, showed a feasible strategy to combine MeJA and SA treatment with transgenic technology for the enhancement of tanshinone, an active diterpene which is widely used in the treatment of cardiovascular diseases, in *Salvia miltiorrhiza* hairy roots [77], also SA was reported to enhance anti-inflammatory activity of *Aloe vera* by increasing its anthraquinones [78].

### 4.1. Phenolic Compounds

Phenolic compounds (more than 8,000 in Nature), can be classified based on the number and arrangement of their carbon atoms in flavonoids (flavonols, flavones, flavan-3-ols, anthocyanidins, flavanones, isoflavones and others) and non-flavonoids (phenolic acids, hydroxycinnamates, stilbenes and others) and they are commonly found conjugated to sugars and organic acids.

Phenolic compound contents have been associated with flavour and colour characteristics of fruits and vegetables. These compounds have additional multiple roles in plants, including attracting insects for seed dispersion and pollination and being part of the natural defense system [79]. Moreover, in recent years, phenolic compounds have been intensively investigated because of their potential health-promoting effects, such as anti-inflammatory [80], antimicrobial [81], antiallergic [82], vascular [83] and cytotoxic antitumor activity [84], but the most cited biological activity is based on their antioxidant capacity, related with its chemical structure that confers them redox properties [85,86]. The accepted wide range of beneficial effects of phenolic compounds initiated, attempts to stimulate their accumulation in crop plants by agricultural technologies. Several reviews summarized the advantages of targeted pre- and post-harvest elicitor treatments to obtain fruits and vegetables enriched with beneficial phytochemicals [87,88,89]. Alfalfa three-day-old sprouts subjected to high-light (700 µmol·m^−2^ s^−1^ for 1 day) and chilling (a growth chamber at 4 °C with a light intensity of 120 µmol·m^−2^ s^−1^ for 1 day) accumulated about 2.0 and 1.5 times, respectively, significantly higher concentration of ferulic acid. Therefore, high-light seems to elicit a stronger response than chilling in enhancing the phytochemical content [43]. The largest accumulation of sinapic acid (by 83% more compared to untreated control) occurred following high-light treatment (700 µmol·m^−2^ s^−1^ for 1 day) in broccoli sprouts, similar to ferulic acid in alfalfa, however, chilling did not seem to have any effect on the sinapic acid content in broccoli sprouts [43]. Examples of biotic and abiotic elicitors affecting different groups of phenolic compounds are listed in Table 2.

**Table 2:** Phenolic compounds increased by elicitors.

Plant Food	Elicitor Treatment	Application	Target Compounds Class and Increase	Reference
“Fuji” apples	Ethephon (2-chloroethyl phosphonic acid) (100 mg/L)	Sprayed for 4 weeks before commercial harvest	Anthocyanins (8-fold), and flavonols (2-fold) during fruit maturation	[90]
Grape berry fruits	Ethanol (5 g/100 mL)	Sprayed for 8–9 weeks after anthesis	Anthocyanins (3-fold)	[91]
Butter Lettuce	JA 1 µM	Sprayed after 21 days of germination	Total phenolics (280%) Flavonoids (133%) Phenolic acids (360%)	[92]
Lettuce cv. “Lollo Rosso”	UV-full range (UV-A and UV-B)	Radiation during cultivation	Flavonoids (130%) and phenolic acids (200%)	[93]
Purple-flesh potatoes	Wounding (vegetable slicer)	After harvest	Total phenolics (60%)	[94]
Strawberry fruits	CO_2_ (ambient + 600 µmol)	28 months	Anthocyanin and flavonols (30%–50%)	[95]
Sweet basil	MeJA 0.5 mM	Sprayed when the plants had five or six leaves	Rosmarinic acid (50%) and caffeic acid (38%)	[96]
Greek oregano	Chitosan oligosaccharides (50 and 200 mg/L)	Sprayed for 2 weeks prior to the anticipated flowering time	Phenolic acids and flavonoids (30%)	[97]
Pea sprouts	Folin acid (50 µM) and vitamin C (500 µM) solutions	Soaking seeds for 12–48 h	Total phenolic compounds (20%)	[98]
Pea sprouts	Folin acid (50 µM) and vitamin C (500 µM) solutions	Soaking seeds for 12–48 h	Total phenolic compounds (20%)	[98]
Olive trees organs	Nutrient solution “Brotomax” (0.3 g/100 mL) (urea nitrogen, copper, manganese and zinc)	Sprayed for 120 days after anthesis	Tyrosol, catechin, and oleuropein (20%)	[99]
Radish sprouts	NaCl (100 mM)	In 0.5% agar media for 3, 5 and 7 days after sowing seeds	Total phenolics (30% and 50% in 5 and 7-days-old sprouts, respectively)	[100]
Radish, chinese kale and pak choi 3-day-old sprouts	Glucose (5 g/100 mL)	Hydroponic system for 3 days after sowing seeds	Total phenolics (20%)	[53]
Broccoli 7-day-old sprouts	Sucrose, fructose and glucose (146 mM)	In 0.5% agar media for 5 days after sowing seeds	Total anthocyanins (10%)	[55]
Broccoli 7-day-old sprouts	Sucrose and mannitol (176 mM)	Hydroponic system for 5 days after sowing seeds	Total anthocyanins (40%) and phenolics (50%)	[101]

Elicitors also have been applied as a complementary treatment to fungicides, such as the exogenous application of benzothiadiazole and MeJA, increasing, at the same time, the flavonoids content (anthocyanin, flavonol, and proanthocyanidin) in grapes and showing higher color intensity and total phenolic content in wines [54].

### 4.2. Glucosinolates

Glucosinolates (GLS) comprise a relatively small but diverse group of over 130 nitrogen and sulfur-containing natural products found almost exclusively in cruciferous plants [102]. The glucosinolate core structure comprises a *β*-thioglucoside *N*-hydroxysulphate, containing a side chain and a *β*-d-glucopyranose moiety [14]. The structure of the side chain is highly variable and determines the glucosinolate classification as aliphatic, indolic, or aromatic [103,104] according to whether their amino acid precursor is methionine, tryptophan, or an aromatic amino acid (tyrosine or phenylalanine), respectively [14]. Glucosinolates are plant defense compounds against various pathogens and pests, and are accumulated preferentially in the organs that contribute most to the growth cycle of the plant [102]. Besides, these compounds have a potential benefit to protect humans against certain cancers, particularly lung and those of the gastrointestinal tract, and also in the reduction of risks for cardiovascular diseases [9,105,106]. However, there are still many areas that need further research to avail the full health benefits of these compounds [107]. Glucosinolates are also responsible of organoleptic properties in some plants, such as cauliflower and mustards [108].

Glucosinolates profiles can be altered by treatments with elicitors [21,109]. Exogenous application of SA, JA and MeJA have been widely studied because of the results in expression of large number of genes involved in resistance responses, among these are genes related to biosynthesis of phytochemicals in plants [110]. SA, JA and MeJA serve as signaling molecules induced by pathogen infestation [24] and mechanical wounding [56]. Treatment of *Brassicaceae* plants with these elicitors can stimulate the increase of glucosinolate content. Baenas *et al.*, (2014), reported that MeJA elicitor (25 µM) was highly effective to increase the total glucosinolates in 5 different 8-day-old *Brassica* and *Raphanus* sprouts, specially, the concentration of the health-promoting glucoraphanin and glucoraphenin by 50% [52].

The individual classes of glucosinolates respond differently to the elicitor treatment. Treatment with SA and MeJA increased the total amount of glucosinolates, particularly levels of aromatic and indole glucosinolates, in secondary roots of turnip, in contrast, SA or MeJA either reduced or did not affect the levels of aliphatic glucosinolates [111]. Kiddle *et al.* reported that JA induces mainly indole glucosinolates in leaves, and the intensity of this “induction” depended on the JA concentration applied and the age of the leaf, retaining developing leaves higher levels than mature leaves [112]. Examples of biotic and abiotic elicitors affecting glucosinolates are showed in Table 3.

**Table 3:** Glucosinolates increased by elicitors.

Plant Food	Elicitor Treatment	Application	Target Compounds Class and Fold Increase	Reference
Brassica 7-day-old sprouts cotyledons and leaves	JA spray (5 nmol)	Topically	3-indolylmethyl GLS (6-fold) in *B.* *napus*; 4-hydroxy-3-indolylmethyl GLS (9-fold) in *B.* *rapa*; both indole GLS (2-fold) in *B. juncea*	[56]
Turnip root exudates	MeJa (130 μM)	Added in the hydroponic system for 10 days	Indole GLS (4-fold)	[113]
Broccoli sprouts	Sucrose (146 mM)	In 0.5% agar media for 5 days after sowing seeds	Total GLS (2-fold)	[55]
Broccoli 7-day-old sprouts	Methionine (5 mM) Tryptophan (10 mM) SA (100 μM) MeJA (25 μM)	Daily exogenous spraying during 3, 5 and 7 days	Aliphatic GLS (30%) Indole GLS (80%) Indole GLS (30%) Indole GLS (50%)	[25]
Radish, chinese kale and pak choi 3-day-old sprouts	Glucose (5 g/100 mL)	Hydroponic system for 3 days after sowing seeds	Gluconapin (150% and 60% in Chinese kale and pak choi, respectively) Glucobrassicanapin (110-fold in pak choi)	[53]
Sauerkraut (*B. oleracea* L. var. capitata)	0.5% NaCl and 0.3 mg of sodium selenite/kg	Added to fresh cabbage before fermentation	Indole GLS hydrolysis products (indole-3- carbinol and indole-3- acetonitrile in 70% and 10%, respectively)	[114]
Radish sprouts	NaCl (100 mM)	In 0.5% agar media for 3, 5 and 7 days after sowing seeds	Total GLS (50% and 120% in 5 and 7-days-old sprouts, respectively)	[100]
*Brassica* 8-day-old sprotuts	MeJA (25 µM) JA (150 µM) Sucrose (146 mM)	Sprayed for 5 days before harvest	Total GLS Broccoli: >50% Turnip: >20% Rutabaga: >100%	[52]
*Raphanus* 8-day-old sprotuts	MeJA (25 µM) SA (100 µM) Glucose (277 mM)	Sprayed for 5 days before harvest	Total GLS: > 20%	[52]
Broccoli 7-day-old sprouts	Sucrose and mannitol (176 mM)	Hydroponic system for 5 days after sowing seeds	Total GLS: > 50%	[101]
Broccoli florets	Ethanol evaporated (500 μL/L)	6 h after harvested	Total GLS: > 50%	[115]
Broccoli florets	MeJA spray (250 µM)	Aerial portions twice per week from flowering to head formation	Indolyl GLS: > 30%	[91,116]

### 4.3. Carotenoids and Betalains

Over the past few years, there has been a surge in interest in fat-soluble compounds, such a carotenoids, and water-soluble compounds, such as betalains, due to their beneficial effects on human health [117]. Carotenoids were initially described as playing a role in the protection against photo-oxidative processes, and they have been extensively studied for the prevention of cancers and cardiovascular diseases and for their photoprotective properties [118].

Tomato fruits cv. Liberto were subjected to UV-B radiation before harvest with an UV-B dosage of 0.075 and 0.15 Wh m^−^^2^ after different adaptation times of 22 and 44 h, the concentrations of carotenoids, lycopene and β-carotene, in ripe tomato fruits were higher increased by an UV-B dosage of 0.075 Wh m^−2^ after 22 h of adaptation time [119].

Betacyanins (red-violet pigments) and betaxanthins (yellow pigments) are a group of chromoalkaloids known as betalains presents in *Caryophyllales*. Interest in betalains is determined by their antiradical activity and their use as additives for food, drugs and cosmetic products. Hydrogen peroxide treatment (sprayed and infliltrated with 0.1%, 0.33% and 1% H_2_O_2_) led to a significant betacyanin accumulation in *Suaeda salsa* L. sprouts, the oxidative stress signal leading to betacyanin production, may be perceived by roots initially, then was transferred to leaves and the signal transduction was performed as betacyanin accumulation induced in leaves [120]. The increase in the microelement Co^2+^ from 1–5 μM also resulted in an 60% increment on the production of betalains, however, Mo^2+^, Fe^2+^ and Cu^2+^ presented a positive (10% increment) but less marked effect, while the increase of Mn^2+^ did not show effects on the production of betalains compared to control medium [121].

### 4.4. Nutrients with Biological Activity

Elicitation of plants has been studied not only to improve the nutraceutical potential of low-processed food, but also the nutritional value (content of vitamins, bioactive peptides and carbohydrates). Vitamins are vital nutrients required by organisms. Vitamin A is essential for normal cell growth, immunological functions and vision, and is found in foods in the form of provitamin-A [122]. Vitamin E, with the α-tocopherol form being the most active in humans, is considered to be one of the most potent lipid-soluble antioxidants *in vivo* [123]. Folate (a collective term used for folic acid and its derivatives) is an important component of vitamin B, which is involved in a number of cellular metabolic processes, mainly playing a role as co-factor in the synthesis of nucleic acids, amino acids, pantothenate and formyl methionine-transfer RNAs [124]. Most recent evidence from a population-based cohort study in Europe lends further support to the notion that an increased intake of folate from food sources, may be associated with a lower risk of pancreatic cancer [125]. Vitamin C, including ascorbic acid and dehydroascorbic acid, is one of the most important nutritional quality factors in many horticultural crops and has many biological activities in the human body, such as the prevention of scurvy, reduction of plasma cholesterol level and as antioxidant, reportedly reduces the risk of arteriosclerosis, cardiovascular diseases and some forms of cancer [126]. Therefore, there is an increasing interest in fortifying many foods with vitamins.

The content of vitamins in fruits and vegetables can be influenced by various factors such as genotypic differences, pre-harvest climatic conditions and cultural practices, maturity and harvesting methods, and postharvest handling procedures [26,127]. Special treatments, including precursor feeding and elicitor application can be used to increase metabolite production. Foliar application (250 µM) of MeJA and SA caused rapid 2-fold increase of folate in coriander (*Coriandrum sativum*) foliage, as well as, treated plants presented higher stability of folates than untreated foliage, during processing and storage [124]. The application of 200, 300 µM of SA and 0.01% chitosan induced increases, by 26%, 18% and 54%, respectively in the content of vitamin C in 5 days old broccoli sprouts [25]. Higher levels of ascorbic acid (in comparison with controls) have been found in 4-day-old lentil sprouts after elicitation with temperature stresses (4 °C and 40 °C for 1 h) [128]. Broccoli sprouts grown in an environment chamber with a 16 h light/8 h dark cycle were found to have much higher concentrations of vitamin C (by 83%) than those grown in the dark [19]. A considerable enhancement on the production of α-tocopherol was observed after the administration of 5 µM JA or by hypoxic conditions both in sunflower and *Arabidopsis thaliana* cell cultures [26]. Folic acid and vitamin C have been also used as exogenous growth enhancers to elicit pea (*Pisum sativum*) seedling vigour and phenolic content. Concentration of 50 μM folic acid and 500 μM vitamin C were optimum to both agronomic and biochemical seed vigour parameters, as well as, the levels of enhanced phenolic content, which were highest on days 8 and 10 of germinating seeds [98].

The starch content has been influenced in lentil sprouts after different germination conditions (elicitation by solution with 100 and 300 mM NaCl), being reduced by 50%, as well as the *in vitro* digestibility and predicted glycemic index of sprouts [129]. Also a decrease in total starch, high content of resistant starch and low starch bioaccessibility, a decrease in protein content and subsequent elevation of non-protein nitrogen fraction was reported in lentil sprouts after a elicitation treatment with H_2_O_2_ [130].

Food-derived bioactive peptides may have regulatory functions in the human system beyond normal and adequate nutrition (such as antimicrobial properties, blood pressure-lowering (ACE inhibitory) effects, cholesterol-lowering ability, antioxidant activities, *etc.*) [131]. As an example, some soy peptides induced the expression of defense genes implicated in phytoalexin production and pathogen defense after treatment of the aerial portion of soybean plants with hormones involved in elicitation [132].

Mineral content also could be affected by elicitation. Salicylic acid (0.5 mM) completely alleviated the negative effects of mustard plants growth under NaCl stress, increasing the uptake of major nutrients such as nitrogen, phosphorus, potassium and calcium [133]. The use of elicitation, based on natural defence mechanisms of plants, allowed the differentiation of food products and production of directed food designed for specific consumer groups (e.g., diabetics, the overweight, Alzheimer’s and cardiovascular disease sufferers, among others).

## 5. Future Trends

The controlled short-time elicitation stresses, during the pre-harvest and post-harvest period, can be used as a tool by the fresh produce industry to obtain healthier products by enhancing their nutraceutical content. Similarly, controlled treatments can be utilized by the food processing and dietary supplement industry as tools to enhance the extractable yields of specific active compounds that have nutraceutical or other functional properties.

Interest in functional foods has been growing over the last decade as consumers become increasingly concerned with diet and nutrition. The industry continues to seek new and unique ingredient and health claims, making the idea of developing more functional food quite compelling. A special emphasis is placed on the biologically active compounds or groups of compounds responsible for the therapeutic applications, and their action mechanisms. Also, the quality and safety regulation of functional products should be established in food industry. Thus, elicitors may be a complementary strategy to breeding programs, production management, or genetic engineering activities. Understanding the interaction among stressors will make possible to find practical applications.

On the other hand, studying elicitor-activated signaling pathways with the purpose of identified signaling components, should be an efficient strategy for activating defense responses in the plant, in order to replace or reduce chemical applications to protect crops [45,110]. .

For new or enhanced plant products, it would be appropriate and unavailable the evaluation of functional properties to demonstrate the potential to obtain safe and effective non-pharmacological alternatives for human health. This may provide a new approach for disease prevention and population wellbeing monitored in clinical trials [134].

## 6. Conclusions

Understanding how plant tissues and their specific secondary metabolic pathways respond to different abiotic and biotic stresses, applied alone or in combinations, would be the basis for designing strategies to enhance phytochemicals in foods. The accurate determination of the effect, driven by the use of the distinct elicitors applied in selected time points of the plant growth, may allow strategies and tools to obtain tailored foods with enhanced health-promoting phytochemicals [69]. The resulting products and ingredients could be considered for functional foods or nutraceutical development that will provide benefits beyond basic nutrition and/or claims for health benefits.
